# Comparison of Automated Breast Ultrasound and Hand-Held Breast Ultrasound in the Screening of Dense Breasts

**DOI:** 10.1055/s-0040-1722156

**Published:** 2021-04-15

**Authors:** Fernanda Philadelpho, Maria Julia Gregorio Calas, Gracy de Almeida Coutinho Carneiro, Isabela Cunha Silveira, Andréia Brandão Ribeiro Vaz, Adriana Maria Coelho Nogueira, Anke Bergmann, Flávia Paiva Proença Lobo Lopes

**Affiliations:** 1 Radiology Department, Diagnósticos da América (DASA), Barra da Tijuca, RJ, Brazil; 2 Clinical Epidemiology Program, Instituto Nacional de Cancer (INCA), Rio de Janeiro, RJ, Brazil; 3 Radiology Department, Universidade Federal do Rio de Janeiro, Rio de Janeiro, RJ, Brazil

**Keywords:** dense breast, screening, hand-held breast ultrasound, automated breast ultrasound, breast cancer, mama densa, rastreamento, ultrassonografia convencional, ultrassonografia automatizada, câncer de mama

## Abstract

**Objective** To compare hand-held breast ultrasound (HHBUS) and automated breast ultrasound (ABUS) as screening tool for cancer.

**Methods** A cross-sectional study in patients with mammographically dense breasts was conducted, and both HHBUS and ABUS were performed. Hand-held breast ultrasound was acquired by radiologists and ABUS by mammography technicians and analyzed by breast radiologists. We evaluated the Breast Imaging Reporting and Data System (BI-RADS) classification of the exam and of the lesion, as well as the amount of time required to perform and read each exam. The statistical analysis employed was measures of central tendency and dispersion, frequencies, Student t test, and a univariate logistic regression, through the odds ratio and its respective 95% confidence interval, and with *p* < 0.05 considered of statistical significance.

**Results** A total of 440 patients were evaluated. Regarding lesions, HHBUS detected 15 (7.7%) BI-RADS 2, 175 (89.3%) BI-RADS 3, and 6 (3%) BI-RADS 4, with 3 being confirmed by biopsy as invasive ductal carcinomas (IDCs), and 3 false-positives. Automated breast ultrasound identified 12 (12.9%) BI-RADS 2, 75 (80.7%) BI-RADS 3, and 6 (6.4%) BI-RADS 4, including 3 lesions detected by HHBUS and confirmed as IDCs, in addition to 1 invasive lobular carcinoma and 2 high-risk lesions not detected by HHBUS. The amount of time required for the radiologist to read the ABUS was statistically inferior compared with the time required to read the HHBUS ( *p* < 0.001). The overall concordance was 80.9%. A total of 219 lesions were detected, from those 70 lesions by both methods, 126 only by HHBUS (84.9% not suspicious by ABUS) and 23 only by ABUS.

**Conclusion** Compared with HHBUS, ABUS allowed adequate sonographic study in supplemental screening for breast cancer in heterogeneously dense and extremely dense breasts.

## Resumo

**Objetivo** Comparar a ultrassonografia convencional das mamas (US) com a ultrassonografia automatizada das mamas (ABUS) no rastreio do câncer.

**Métodos** Realizamos um estudo transversal com pacientes com mamas mamograficamente densas, sendo avaliadas pela US e pela ABUS. A US foi realizada por radiologistas e a ABUS por técnicos de mamografia e analisada por radiologistas especializados em mama. A classificação *Breast Imaging Reporting and Data System* (BIRADS) do exame e das lesões o tempo de leitura e de aquisição foram avaliados. A análise estatística foi realizada através de medidas de tendência central, dispersão e frequências, teste t de Student e regressão logística univariada, através do odds ratio, com intervalo de confiança de 95%, e com *p* < 0,05 sendo considerado estatisticamente significante.

**Resultados** Foram avaliadas 440 pacientes. Em relação às lesões, a US detectou 15 (7,7%) BI-RADS 2, 175 (89,3%) BI-RADS 3 e 6 (3%) BI-RADS 4, das quais 3 foram confirmadas, por biópsia, como carcinomas ductais invasivos e 3 falso-positivos. A ABUS identificou 12 (12,9%) BI-RADS 2, 75 (80,7%) BI-RADS 3 e 6 (6,4%) BI-RADS 4, incluindo 3 lesões detectadas pela US e confirmadas como carcinomas ductais invasivos, além de 1 carcinoma lobular invasivo e 2 lesões de alto risco não detectadas pela US. O tempo de leitura dos exames da ABUS foi estatisticamente inferior ao tempo do radiologista para realizar a US ( *p* < 0,001). A concordância foi de 80,9%. Um total de 219 lesões foram detectadas, das quais 70 por ambos os métodos, 126 observadas apenas pela US (84,9% não eram lesões suspeitas no ABUS) e 23 apenas pela ABUS.

**Conclusão** Comparado à US, a ABUS permitiu adequado estudo complementar no rastreio do câncer de mamas heterogeneamente densas e extremamente densas.

## Introduction

Breast cancer is considered one of the most frequent malignancies in women worldwide. 1 A key factor for breast cancer patients is the early detection of the disease as it may improve the outcomes (treatment success and mortality reduction). 2 The workflow of breast screening for this purpose is already known, with mammography being considered the standard screening method. 2 3 4 There are some differences among review boards around the world regarding when to start the screening with mammography. The American College of Radiology Cancer Society and College of Surgeons recommend it for women over 40 years old. 5 The European guidelines on breast cancer screening and diagnosis suggests mammography screening every 2 or 3 years in women over 45 years old. 6 Brazillian Societies (radiology, mastology and gyneco-obstetritics) 3 recommend breast screening with mammography from the age of 40 and also support the recommendation of complementation of the screening with ultrasound (US) in high-density breasts.

Even though it is recommended worldwide as the gold standard screening method, mammography has several limitations, especially that is not equally effective in all women due to different patterns of breast density. 2 It is well described that the sensitivity of the mammogram to detect lesions decreases significantly the higher the breast density, a phenomenon known as “masking.” “Masking” occurs as a consequence of the reduced contrast between dense breast tissue and a lesion, and a greater superimposition of tissue that might lead to misdiagnosis. 4 The overall sensitivity of mammography as a screening method is 85%. However, in women with dense breast tissue, its sensitivity is reduced to between 47.8 and 64.4%. 2 7 High density breast tissues tend to decrease with age; 8 however, in up to 50% of women, this may be a life-long issue. 2 4 7 9

There are several risk factors associated with breast cancer, including genetic factors, age, behavioral factors (smoke, diet, among others), family history, hormone factors, and mammographic breast density. In some series, it was observed that women with extremely high-density breasts have more probability to develop cancer when compared with those with low-density breasts. 7

For this reason, alternative screening tools are needed for the correct evaluation of these patients. Breast US is well recognized as a diagnostic tool; however, it is not usually used for screening purposes in all women. It is specific valuable in the case of patients with high-density breasts, especially when it is performed by an experienced professional. 10 11 12

When thinking of an algorithm to be stablished in the screening of patients with high-density breasts, multiple observational and retrospective studies support the use of US as a supplemental screening tool to detect breast lesions. 4 10 11 13 In Brazil, there is an increased demand for hand-held breast ultrasound (HHBUS) as a screening tool, since its use is recommended by our national guidelines as a complementary exam for high-density breasts. 3 Due to this increased demand, there are not enough experienced radiologists specialized in breast imaging to perform the exams. Most of the time, the exam is performed by a general radiologist, with less experience in breast imaging, resulting in reduced sensitivity and increased false positives, when compared with the specialist in breast exams. This is partially because general radiologists are neither experienced in reading mammography nor familiar with the American College of Radiologists Breast Imaging Reporting and Data System (ACR BI-RADS). 14

One of the main challenges with HHBUS is to ensure reproducible and standardized images and interpretation, as it is a highly operator-dependent technique and is also dependent on the experience of the performer. 10 15 Another issue that must be taken into account is that HHBUS can be too time consuming for the radiologist, especially when the demand is high. 16

The automated breast ultrasound (ABUS) has been approved by the Food and Drug Administration (FDA) in 2012 and has been widely used as a supplemental screening tool for breast cancer. It was designed to supplement some of the issues regarding HHBUS, such as operator dependency, little experience with breast exams, and low sensitivity and reproductibility. It is supposed to be used as an adjunct to mammography for screening asymptomatic women with dense breasts. 15 17 18

One potential advantage of ABUS is also the possibility to divide acquisition and interpretation, while still being effective. 17 18 19 A main advantage of the ABUS is that it may be performed by a trained person without loss in the performance as its automated acquitision provides proper orientation, full breast volume images with great reproductibility, and also detectability. 4 17 By using ABUS, the breast radiologist can focus on the interpretation, as the entire process is conducted by other health personnel, thus improving the exam workflow; and the specialized radiologist can dedicate his full time to diagnosis. For those who will perform ABUS, it is a simple method that does not require a lot of training. 17 20

Another advantage point is that the acquired data (including 3D volume) can be evaluated further at anytime and independently by two different radiologists (double-reading), which is useful in cases of doubt and also for use in clinical trials. 15 In several studies, ABUS had results similar to those of HHBUS regarding detection of occult breast lesions. 4 19

The aim of the present study is to compare the performance of HHBUS and ABUS in our setting as a supplemental screening tool for breast cancer.

## Methods

A unicentric cross-sectional study was performed in our private imaging institution after approval by our national Institutional Review Board (CAAE: 58146816.3.0000.5257) and written informed consent obtained from all participants.

Asymptomatic women who had heterogeneously or extremely dense breast tissue (classified by BI-RADS as C or D) and who underwent screening digital mammography and HHBUS examinations were asked to participate in the study. Mammography exams were used to assure dense breast (BI-RADS as C or D) independently of further BI-RADS classification. After routine exams (mammogram and HHBUS), patients had ABUS examination performed on the same day. Mammography images and reports were available for the radiologists to make sure about the breast density (heterogeneously or extremely dense breast tissue) when interpreting HHBUS or ABUS. When interpreting ABUS or HHBUS, they were blinded to the results of each other. When a suspicious or dubious finding was observed in the ABUS evaluation, another HHBUS exam was performed by a radiologist not involved in the study (considered a recall) to decide whether or not to proceed with biopsy, guided by the HHBUS findings as ABUS cannot be used to guide biopsies. Patients with breast surgery for breast cancer or benign causes (including breast implants) or breast radiotherapy in the previous 12 months were excluded from the study. All images were reported with the current ACR BI-RADS classification. 14 We compared and classified each observed lesion with BI-RADS classification, regarding its features (morphologic characteritics, size and location).

The HHBUS exams were performed by 30 radiologists, some were specialized in breast imaging ( *n* = 13), while all others were not. Variable ultrasound systems were used, all equipped with a linear-array transducer with a bandwidth of 7 to 14 MHz. The mean time to perform HHBUS from beginning to end, observed in the HHBUS machine, was also measured. When an ABUS recall was needed for further investigation, this additional time to perform HHBUS was not taken into account.

**Fig. 1:**
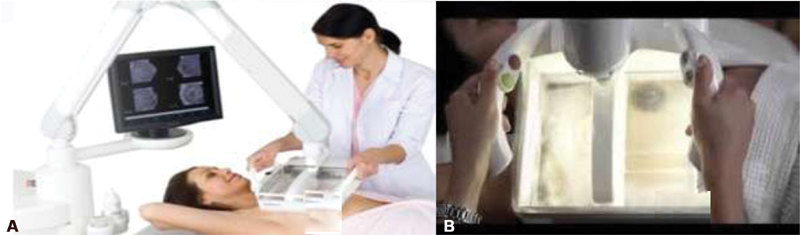
Automated breast ultrasound scanning unit (A) and transducer (B). Patient lies in a supine position with the arms above the head and the technician performs the study, using a 15 cm long transducer with slight pressure.

**Fig. 2:**
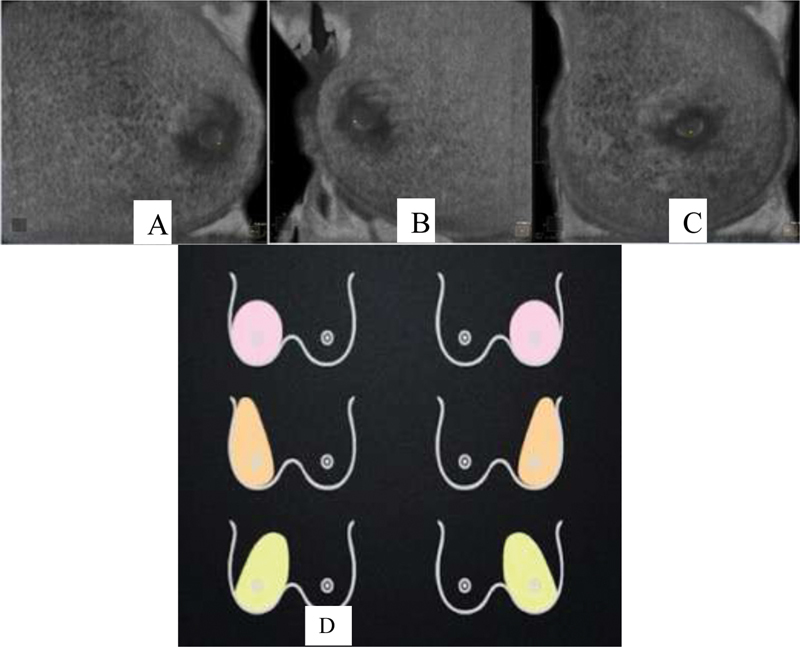
Automated breast ultrasound acquires images (A- lateral view, (B) medial view; and (C) anteroposterior view) and schematic drawings (D) of automated breast ultrasound views: Lateral (orange), medial (yellow), and anteroposterior (pink).

Automated breast ultrasound exams (Invenia ABUS, Automated Breast Ultrasound System, GE Healthcare, Sunnyvale, CA, USA) were performed by one of four trained mammography technicians, with a preestablished protocol. The ABUS system consists of a scanning unit (with a 10–15 MHz high-frequency linear transducer) and the image review workstation. To be performed, the patient lies in a supine position with the arms above the head ( Fig. 1A ). The technician performing the study is only required to apply gel to the breast, and to put the scanning plate and transducer with slight pressure and select the patient's breast size. The breast tissue should be fully covered to avoid air bubble formation on the contact surface. The system then sets all scanning parameters. The transducer slides continuously over a membrane, which is kept in contact with the breast ( Fig. 1B ). The number of required scans to image the whole breast is determined by patient's breast size and ranges from three to four scans per breast. Anteroposterior, medial, and lateral views are routinely acquired ( Fig. 2 ). If there are additional indications, such as for large breasts, superior and inferior views are also acquired. All views must contain the nipple as a reference point, which is marked by the operator at the end of each scan, to allow correct orientation and postprocessing reconstructions. In the anteroposterior view, the nipple should be centered on the image. In the medial and lateral views, the nipple should be at the periphery of the image. The images are acquired with a 15-cm field of view for review. The participating technicians and radiologists received standardized ABUS training, provided by the system vendor, for 1 month, performing 50 exams during this period (data not shown). After acquisition, the axial image series is sent to a dedicated workstation and then can be examined in multiplanar reconstructions, including sagittal and 2-mm-thick coronal images, parallel to the chest wall. All ABUS exams were interpreted by one of the six breast radiologists that participated in the study. The mean acquisition time to perform ABUS by technicians from the time the scan effectively started and finished, observed in ABUS machine, and mean interpretation time to read ABUS by radiologists, from the time the study was opened until the final conclusion, were measured.

We also assessed the exams limitations and presence of pain during ABUS. We divided the perception of pain, as described by the patients, in four categories: absent (no pain), minimal, mild, and severe.

For patients in the BI-RADS 4 category, biopsy results were obtained for comparison. Demographic and clinical characteristics are presented through measures of central tendency and dispersion (quantitative variables) and absolute and relative frequency (qualitative variables). The Student t-test was used to evaluate the difference between the means. Association between breast lesion and exam was performed using a univariate logistic regression, through the odds ratio and its respective 95% confidence interval. Agreement between the methods was obtained by simple concordance. For all comparisons, a statistically significant difference was considered when *p* < 0.05.

## Results

Between August 2017 and July 2018, we enrolled a total of 444 asymptomatic women that were referred for screening mammogram report (classified as BI-RADS as C or D regarding breast density), after they accepted and signed an informed consent to participate in the study. Four patients were excluded from the study due to important limitations during ABUS exams. The clinical and epidemiological characteristics of the patients included in the study ( *n* = 440) are shown in Table 1 . Most patients had heterogeneously dense breasts (95%).

**Table 1:** Clinical and imaging data

Characteristic	HHBUS N (%)	ABUS N (%)
Age (yr)		
Median	48	
Range	20–79	
Breast parenchyma at mammography	
Heterogeneously dense (C)	418 (95)	
Extremely dense (D)	22 (5)	
Pain (during ABUS)		
Absence	–	292 (66.4)
Minimal	–	83 (18.9)
Mild	–	42 (9.5)
Severe	–	23 (5.2)
Final exams BI-RADS		
1 (negative)	153 (34.8)	189 (42.9)
2 (benign)	188 (42.7)	201 (45.7)
3 (probably benign)	96 (21.8)	46 (10.5)
4 (suspicious)	3 (0.7)	4 (0.9)

Abbreviations: ABUS, automated breast ultrasound; BI-RADS, Breast Imaging Reporting and Data System; HHBUS, hand-held breast ultrasound.

Regarding HHBUS, 99/440 (22.5%) exams were performed by breast radiologists ( *n* = 13), who took an overall 7 minutes and 45 seconds (range, 2–27 minute) to perform the exam. The other exams (341/440 [77.5%]) were performed by non-specialized radiologists ( *n* = 17), who took an overall of 4 minutes and 15 seconds (range, 1–33 minute). The mean exam time was 5 minutes and 3 seconds ( *p* < 0.001).

Regarding ABUS, the mean exam acquiring time was 14 minutes (range, 6–24 minute), and the breast radiologist ( *n* = 6) mean reading time was 4 minutes and 25 seconds (range, 2–20 minute). In 68.4% of the exams, 6 views were acquired (3 for each breast); 7 views were acquired in 11.1% of exams (3 for one breast and 4 for the other), and in the remaining 18.4% of exams, 8 views were performed (4 for each breast).

The majority of the patients described no pain (66.4%) or minimal pain (18.9%) during ABUS exam ( Table 1 ). When pain was present, the medial view was the main site (9.5%).

When performing the ABUS exam, we experienced difficulties (limitations) during acquisition in 115/444 (25.9%) of the cases. The most common limitations were acoustic shadowing artifacts, firm breasts (leading to difficulties on the probe positioning), and large breasts ( Table 2 ). We had to exclude 4/115 (2.8%) exams (as previous mentioned) due to important acoustic shadowing artifacts related to lack of adequate breast compression.

**Table 2:** Limitations during automated breast ultrasound exam, related by technicians

Limitations	Patients
Artifacts	26 **
Firm Breast	24
Large breast	20
Small breast	16
Protruding sternum	15
Flabby breast	14
Total	115 (25.9%)

** 4 patients were excluded due to major artifacts (lack of adequate breast compression).

Recall for additional HHBUS due to doubts ( *n* = 4) or suspicious lesion ( *n* = 6) during ABUS exam was only needed in 10/440 (2.27%) exams. In all recalls due to doubts, they occurred in the first months of the study; the other recalls were from BI-RADS 4 findings.

In Table 1 , we also show the overall distribution of BI-RADS classification obtained by HHBUS and ABUS for each exam. Both methods found out more BI-RADS 1 or 2 exams. Regarding the comparison between lesions classified by BI-RADS, in Table 3 we summarized the main findings. Hand-held breast ultrasound detected 15 BI-RADS 2 masses and ABUS 12 BI-RADS 2 masses. We observed 175 lesions BI-RADS 3 with HHBUS; from those, 4 were clustered microcysts, 2 ductal ectasias, and the other lesions ( *n* = 169) were solid masses. With ABUS, 75 lesions were BI-RADS 3; from those, 75 were solid masses. Both methods detected 6 lesions each BI-RADS 4.

**Table 3:** Breast Imaging Reporting and Data System lesions obtained by hand-held breast ultrasound and automated breast ultrasound exams

Lesions and BI-RADS	HHBUS N (%)	ABUS N (%)
Median (range) lesions sizes	0.86 cm (0.3–3.8 cm)	1.13 cm (0.5–3.0 cm)
BI-RADS 2 masses	15 (7.7)	12 (12.9)
BI-RADS 3 lesions	175 (89.3)	75 (80.0)
Solid masses	169 (86.3)	75 (80)
Clustered microcysts	4 (2)	0
Ductal ectasias	2 (1)	0
BI-RADS 4 masses	6 (3)	6 (6.4)

Abbreviations: ABUS, automated breast ultrasound; BI-RADS, Breast Imaging Reporting and Data System; HHBUS, hand-held breast ultrasound.

Considering lesion detection rates, HHBUS showed 126 lesions not seen by ABUS. Automated breast ultrasound detected 23 lesions not seen by HHBUS, and 70 lesions were seen by both methods. No significant difference was observed in the average size of the lesions detected by both methods—HHBUS 1.17 cm *versus* ABUS 1.14 cm ( *p* = 0.662). The overall concordance between the two methods was 80.9%. The lesions missed by ABUS were not suspicious; ∼ 85% (107/126) of them were cysts, fat lesions or normal ducts. From the others misdiagnosed lesions by ABUS, one lesion was near the axilla (measuring 1.0 cm) and 18 lesions had an average of 0.6 cm (range, 0.4–1.0 cm) in both breasts (data not shown).

**Fig. 3:**
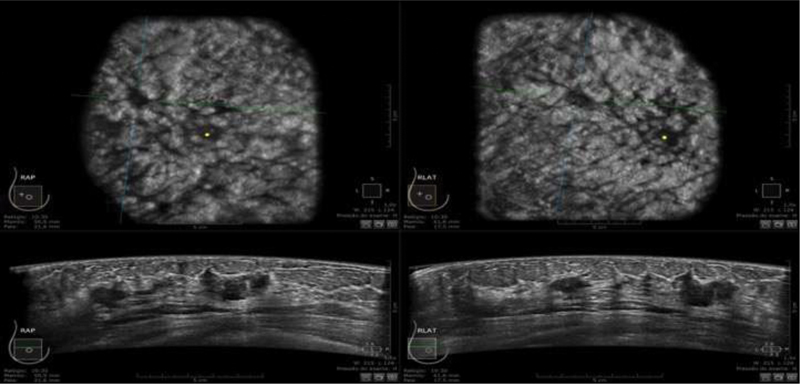
Female, asymptomatic, 65-year-old patient. Automated breast ultrasound exam. Coronal (upper) and longitudinal (bottom) images shows three hypoechogenic, irregular and spiculated masses in the right breast, also detected by Hand-held breast ultrasound. The lesion was classified as Breast Imaging Reporting and Data System 4. Histopathological findings confirmed malignancy - grade 2 infiltrating ductal carcinomas.

**Fig. 4:**
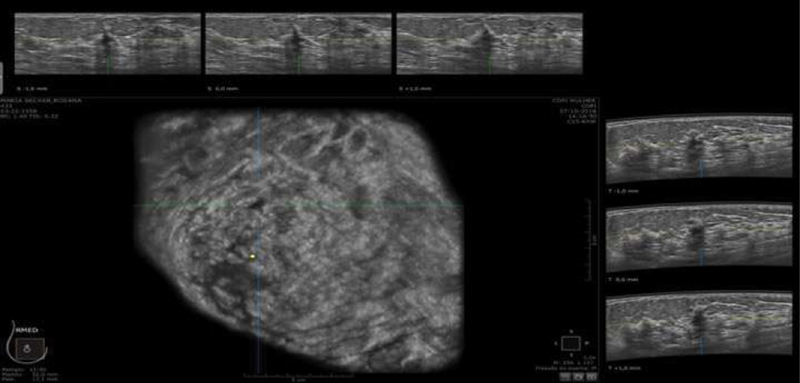
Female, asymptomatic, 60-year-old patient. Automated breast ultrasound showed a hypoechogenic, irregular and indistinct mass in the right breast – classified as a Breast Imaging Reporting and Data System 4 lesion. Histopathologic result confirmed an infiltrating lobular carcinoma.

Lesions classified as BI-RADS 4 were detected by each method in six cases ( Table 3 ). Three were invasive ductal carcinomas (IDCs) in the same patient, correctly described by both methods ( Fig. 3 ). Hand-held breast ultrasound also described 3 other BI-RADS 4 lesions that were BI-RADS 3 or 2 by ABUS; 2 of them were fibroadenomas, and 1 was an inflammatory cyst. So, HHBUS resulted in 50% false positives. Meanwhile, ABUS also described 3 additional BI-RADS 4 lesions not seen by HHBUS, one malignant ( Fig. 4 ) and two high-risk lesions ( Table 4 ). In our study, the cancer detecion rate was 4.5 per 1,000 women for ABUS (all performed by breast radiologists) against 2.3 per 1,000 women for HHBUS (performed by breast radiologists and non-specialist radiologists).

**Table 4:** Breast Imaging Reporting and Data System 4 lesions: comparison between hand-held breast ultrasound, automated breast ultrasound, and histopathology results

HHBUS	ABUS
3 spiculated masses (invasive ductal carcinomas)	3 spiculated masses (invasive ductal carcinomas)
1 complex mass (fibroadenoma)	Described as solid mass, BI-RADS 3
Not diagnosed by HHBUS	1 solid spiculated 1.3 cm mass (radial scar)
1 solid palpable 3.6 cm mass (fibroadenoma)	Described as solid mass, BI-RADS 3
Not diagnosed by HHBUS	1 solid intraductal 0.7 cm mass (papilloma)
1 complex 1.3 cm mass	Inflammatory cyst, described as BI-RADS 2
Not diagnosed by HHBUS	1 solid irregular 0.9 cm mass (invasive lobular carcinoma)

Abbreviations: ABUS, automated breast ultrasound; BI-RADS, Breast Imaging Reporting and Data System; HHBUS, hand-held breast ultrasound.

## Discussion

The main concern for the research of new technologies in breast cancer screening is based on the increasing number of cases worldwide and the limitations of mammography, especially when it comes to dense breasts. Several reports show that US may be a useful complementary screening method for women with dense breast tissue to detect occult breast lesions. 4 10 11 13 20 Unfortunately, even though US is an extremely valuable tool and is recommended as a supplemental imaging method, it is an operator-dependent method associated with low sensitivity and false positives when performed by inexperient personnel, thus leading to unecessary biopsies in the case of misdiagnosis. 19 Therefore, ABUS comes as an important alternative tool to overlap these technical issues regarding experience and operator-dependent methods, as it can be mainly interpreted by breast radiologists. 20

Hand-held breast ultrasound is the routine supplemental screening technique. It has the major advantage of not using radiation, and it allows for a detailed evaluation of an abnormality. You can also add more details about a potential lesion using color Doppler and elastography to establish the accurate diagnosis. 10 20 In Brazil, the use HHBUS is a common screening method, as an adjunct method to mammography for asymptomatic women with dense breast tissue. However, most of the time, the operator is a general radiologist, with low experience in breast imaging, which might lead to misdiagnosis or overdiagnosis of lesions. 3 10 11 In our practice, and as reflected in the present study, ∼ 77.5% of the HHBUS were performed by non-specialiazed radiologists in breast imaging, thus reducing cancer detection and increasing false positives. In addition to a lack of experience in breast imaging, the poor quality of HHBUS exams performed by radiologists non-speciliazed in breast imaging may be accentuated by the shorter time taken to perform the exam, which was statistically inferior when compared to the amount of time taken for specialized radiologists to perform the exam, in our study.

Automated breast ultrasound comes as a new imaging technology for automatic breast scanning with US to overlap the main limitations of HHBUS. Moreover, in countries like Brazil, where only physicians are allowed to perform clinical ultrasounds, ABUS allows acquisition by non-physician personnel. As previously mentioned, it also has the advantage of decoupling acquisition and interpretation, with the possibility of double-reading and objective comparison with previous exams. 21 Another benefit is the ability to document the entire breast volume and to provide 3D images, thus reducing potential misdiagnosis of lesions, and, as the radiologist will be focused only on the interpretation of the findings, it may improve diagnostic rates. 22

Regarding the amount of time required for ABUS reading, several studies have reported it. 21 23 24 25 Skaane et al. 25 showed that the mean interpretation time was ∼ 9 minutes for a bilateral examination. In our study, the reading time of ABUS was shorter, compared with that reported in Skaane's study, at ∼ 4 minutes and 25 seconds for both breasts.

In relation to scan time, as in other studies, HHBUS took less time to be performed compared with ABUS, considering the time from start to finish. It is important to highlight that, unfortunately, we had some outlier results considering HHBUS scan time. Some operators took an extremely short amount of time to perform it, which, in turn, influenced our results to lower the average time when compared with the literature; this could also influence the proper evaluation of the exam. Lin et al. 26 reported an average ABUS scanning time of 11.9 minutes compared with an average HHBUS scanning time of 6.8 minutes. In our study, ABUS scanning time took an average of 14 minutes. However, the main advantage of ABUS, in our point of view, is the possibility of being performed by a technician rather than the physician, who can then use his/her entire time to read the exam.

The use of both HHBUS and ABUS has been described to improve detection of small invasive cancers in women with dense breasts, compared with screening with mammography. 27 An increase in cancer detection rate (CDR) has also been described when HHBUS is performed during the screening. 16 20 27 28 Automated breast ultrasound is also described as a supplemental screening method that can improve CDR. 19 29 Although some studies 15 26 30 31 32 compare ABUS and HHBUS regarding CDR, these reports are based on relatively small populations and focus primarily on the diagnostic setting. They show similar performance of ABUS and HHBUS for detection and diagnosis of breast lesions. Most studies using the new-generation ABUS scanners reported a high sensitivity and specificity, comparable to, or sometimes better than, HHBUS. 15 26 30 31 32 Specifically, Lin et al. 26 reported optimal agreement between ABUS and HHBUS as well as between ABUS and the results of pathologic diagnosis. A recent meta-analysis by Meng et al. 33 revealed a 92% (range: 89.9–93.8%) pooled sensitivity and an 84.9% (range 82.4–87%) specificity of ABUS, with no significant difference between ABUS and HHBUS in terms of diagnostic accuracy. Our study compared ABUS and HHBUS with focus on the screening setting, and the cancer detecion rate was 4.5/1,000 women for ABUS against 2.3/1,000 women for HHBUS, similarly to previous studies; however, we have to take into account that all ABUS exams in our study were interpretated by breast radiologists, which may interfere with the higher detection rates.

As with other new technologies, the learning curve is always an issue and must be taken into account when evaluated as well as when implemented for screening purposes. It is well known that when you have an operator-dependent device, accurate training is a key factor for the results, especially regarding the sensitivity and specificity of the method. 25 This is well illustrated by Arleo et al., 34 who observed that after implementation of ABUS, they experienced a drop out in recalls over time from the first month of use (24.7%) to the third (12.6%). Despite the possibility that all ABUS exams may be read mainly by breast radiologists, specific training is still required, as any other new technology, especially to avoid pitfalls. 17 21 We had a low recall rate (2.27%), and it was observed on the first scanned patients, for the doubt lesions or due to suspicious lesions, probably due to our learning curve. With technicians' learning curve, exams presented less artifacts and, with radiologists' learning curve, it was easier to differentiate an artifact from a real lesion. Hence, radiologists and technologists need to be familiar with these image artifacts and how to reduce them.

Hand-held breast ultrasound presented more than twice as many BI-RADS 3 lesions compared with ABUS, resulting in increased US follow-up scans. The increase in reported probably benign lesions, instead of benign lesions or negative exams, is most likely explained by the variability in the HHBUS operator's experience, which is a known disadvantage of the method.

The mean diameter of the lesion is an important factor in lesion detectability for ABUS, as it may misdiagnose small lesions. 30 31 32 This represents a limitation of the technology itself, but it is also observed with HHBUS. However, Wang et al. 32 reported a higher diagnostic accuracy of ABUS compared with HHBUS for lesions smaller than 1 cm. Other investigators suggested lower detection rates for benign lesions compared with malignant lesions, with ABUS having lower diagnostic accuracy compared with HHBUS for lesions with a benign appearance and also regarding BI-RADS category. 30 In the present study, the misdiagnosed lesions by ABUS measured 0.6 cm in average, and all of them were benign or probably benign lesions, as described in previous studies.

Regarding the clinical applications of ABUS, this technique was first indicated as a screening method in an effort to improve breast cancer diagnosis, especially in mammographically dense breasts. Due to promising results observed in the screening scenario, it was studied as a diagnostic method besides the screening and for evaluation of tumor response to neoadjuvant chemotherapy, as well as being an additional US method after magnetic resonance imaging doubts.

The major limitations of the present study were a single-site study with a small number of patients for a screening study, thus reducing the statistical power. Moreover, each patient's image was read by one radiologist without a second look from another radiologist; therefore, interobserver variability was not determined. Besides that, ABUS and HHBUS were not performed and analyzed by the same radiologist, and the majority of HHBUS were performed by non-specialists, differently from ABUS, in which all exams were performed by breast radiologists. Other limitations were that we did not follow up the lesions BI-RADS 3 in the exams and that we had some scan time outliers (with extremely short time to perform the scan in the HHBUS) in some exams.

Future improvements in ABUS, such as optimal parameter adjustment, whole-breast Doppler, and elastography, are under invetigation and may provide further advantages in the screening and diagnosis. 27 28 Also, the integration of ABUS with tomosynthesis may allow formation of hybrid images, which can provide multimodal data for potential classification of breast lesions. 29 Lately, other features for ABUS technology are being developed, and different computer-aided detection (CAD) systems have been added to the device. 21 35

## Conclusion

Compared with HHBUS, ABUS allowed for effective ultrasonographic performance in supplemental screening for breast cancer. With ABUS, the breast radiologists optimized their time, being able to read more exams in less time, and there was a reduction in the detection of probably benign lesions and the need for unecessary follow-up and biopsies. Automated breast ultrasound is a reliable and reproducible tool as a complementary breast screening method. However, radiologists must become familiar with ABUS images to accurately characterize and classify lesions. A learning curve and specific limitations exist; hence, a specific training is required, regardless of the examiner's experience with HHBUS. Besides that, the financial aspect must be taken into account due to the higher costs of the method; however, ABUS is expected to improve the detection of lesions, which, in a final analysis, may also end up saving money due to possible early detection and, therefore, early treatment implementation.
